# Mechanism and Utilization of Ogura Cytoplasmic Male Sterility in Cruciferae Crops

**DOI:** 10.3390/ijms23169099

**Published:** 2022-08-13

**Authors:** Wenjing Ren, Jinchao Si, Li Chen, Zhiyuan Fang, Mu Zhuang, Honghao Lv, Yong Wang, Jialei Ji, Hailong Yu, Yangyong Zhang

**Affiliations:** 1 Institute of Vegetables and Flowers, Chinese Academy of Agricultural Sciences, Key Laboratory of Biology and Genetic Improvement of Horticultural Crops, Ministry of Agriculture, Beijing 100081, China; 2 State Key Laboratory of Crop Genetics and Germplasm Enhancement, College of Horticulture, Nanjing Agricultural University, Nanjing 210095, China

**Keywords:** cruciferous vegetables, Ogura CMS, sterility gene, fertility restorer gene (FR gene), cytoplasmic male sterility, distant hybridization

## Abstract

Hybrid production using lines with cytoplasmic male sterility (CMS) has become an important way to utilize heterosis in vegetables. Ogura CMS, with the advantages of complete pollen abortion, ease of transfer and a progeny sterility rate reaching 100%, is widely used in cruciferous crop breeding. The mapping, cloning, mechanism and application of Ogura CMS and fertility restorer genes in *Brassica napus*, *Brassica rapa*, *Brassica oleracea* and other cruciferous crops are reviewed herein, and the existing problems and future research directions in the application of Ogura CMS are discussed.

## 1. Introduction

**Figure 1:**
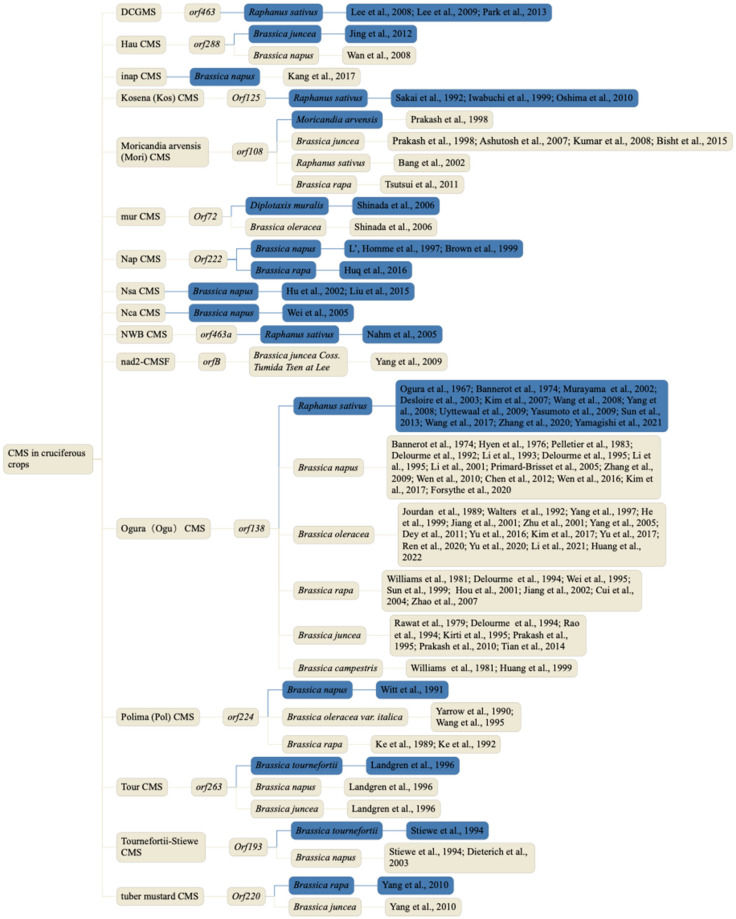
CMS systems reported in cruciferous crops. Blue indicates the origin of a cruciferous vegetable crop [22,23,26,27,28,29,30,31,32,33,34,35,36,37,38,39,40,41,42,43,44,45,46,47,48,49,50,51,52,53,54,55,56,57,58,59,60,61,62,63,64,65,66,67,68,69,70,71,72,73,74,75,76,77,78,79,80,81,82,83,84,85,86,87,88,89,90,91,92,93,94,95,96,97,98,99,100,101,102,103,104,105,106,107,108,109,110,111,112].

In 1973, the German botanist Joseph Gottlieb Kolreuter first observed the phenomenon of male sterility (MS). Researchers defined MS as plant failure to produce dehiscent anthers, functional pollen or energetic male gametes [1,2]. To date, researchers have observed MS in 43 families, 162 genera and approximately 617 species, including *Zea mays*, *Oryza sativa*, *Raphanus sativus*, *Gossypium hirsutum*, *Allium cepa*, *Sorghum bicolor* and *Glycine max* [3,4,5,6]. According to different inheritance characteristics, MS can be divided into genic MS (GMS) and cytoplasmic MS (CMS). GMS is controlled by one or more nuclear genes, while CMS is usually controlled by mitochondrial genes and a few nuclear genes. CMS has been found in more than 150 higher plants [7,8]. Because female organ function is not affected by CMS and CMS lines can be leveraged to maximize cost-effectiveness, the CMS/FR system has become the most widely used MS system in cruciferous crops [9,10]. To date, numerous different CMS systems have been reported in cruciferous crops [11], including Ogura CMS (Ogu CMS), Nap CMS, Polima CMS (Pol CMS), Kosena CMS (Kos CMS), Tour CMS, Moricandia arvensis CMS, Nsa CMS, Nca CMS, Hau CMS and inap CMS (Figure 1). In addition, several CMS-related genes have been identified, including *orf138* of CMS-Ogu in *Raphanus sativus* [12,13,14,15,16,17,18,19,20,21], *orf263* of CMS-Tour in *Brassica tournefortii* [22], *orf224* of CMS-Polima, *orf222* of CMS-Nap in *Brassica napus* [23,24], *orf220* of a new type of CMS line and *orf288* of CMS-hau in *Brassica juncea* [25,26,27] (Figure 1). Among all types of CMS, Ogura CMS is the most widely applied in cruciferous crops due to its significant advantages of stable sterility and complete abortion.

## 2. Mapping and Cloning of the Ogura CMS Gene

### 2.1. Identification of the Ogura Sterility Gene orf138

The determination of the sterility gene of the CMS system is a critical step in mechanistic research. Through the transcription pattern profiling approach, glutathione S-transferase (GST) fusion protein experiments and mitochondrial genome sequencing, the *orf138* gene was identified as the sterility gene of Ogura CMS. *orf138* was first identified because the transcription patterns of three mitochondrial structural genes (*atp6*, *atpA* and *coxI*) changed in Ogura CMS radish, but these changes were not strictly correlated with Ogura CMS or Ogura nuclear restorer genes [12,13]. An Ogura-specific mitochondrial DNA fragment containing two ORFs, *orf138* and *orf158*, was subsequently reported, which was consistent with the sterility phenotype in the 13 somatic hybrids of Ogura CMS *B. napus* obtained from protoplast fusions [14,15]. Sequence analysis and transcript analysis of this region showed that there were also transcripts of *orf158* in fertile plants but no transcripts of the *orf138* gene in fertile revertant plants. Therefore, it was speculated that the gene controlling Ogura CMS is the mitochondrial gene *orf138* [16]. In addition, a polypeptide confirmed to be the product of the *orf138* gene using antibodies against a glutathione S-transferase-ORF138 fusion protein was only found in sterile-plant mitochondria isolated from *B. napus* with Ogura CMS, which further confirmed that *orf138* was the sterility gene in Ogura CMS [17,18]. The mitochondrial genome sequencing of the Ogura CMS line and maintainer line promoted the identification of the Ogura CMS gene *orf138*. The unique presence of the *orf138* gene in Ogura CMS lines was confirmed by comparing the mitochondrial genomes of Cruciferae crops, and the key role of the MS-causing gene *orf138* was identified. The Ogura CMS mitochondrial genomes were generally larger and showed higher recombination and rearrangement than the mitochondrial genomes of the maintainer line, leading to the loss or generation of some genes (Table 1). For example, the mitochondrial genome sequencing of Ogura CMS radish showed that the mitochondrial genome had four special regions compared with that of normal fertile plants and that the *orf138* gene was located at the edge of the largest region (15,255 bp), which was occupied by the *cox1* gene in normal fertile radish [18,19,20]. Meanwhile, Ogura CMS radish possesses an additional copy of the genes *atp9* and *trnfM*.

### 2.2. Functional Verification of the Sterility Gene orf138

The function of *orf138* was verified through transient gene overexpression in *Arabidopsis thaliana* and knockdown approaches. The method of using mitochondrion-targeting peptides to transform sterility genes into the mitochondrial genome has been successfully applied in the functional verification of CMS genes in *G. max*, *O. sativa*, *Beta vulgaris*, *B. napus*, *B. juncea*, *Capsicum annuum* and other crops [50,117,118]. Zhang et al. (2015) cloned the *orf138* gene from the mitochondria of the Ogura CMS line in cabbage, constructed a mitochondrion-targeting expression vector driven by the CaMV35S promoter, transformed it into *A. thaliana* to induce MS, and verified the sterility-related function of the *orf138* gene [119]. Evidence suggests that stable mitochondrial transformation is currently lacking, while recent advances in gene editing have made it possible to easily knock out a gene. The 3′ end of the *orf138* gene has three consecutive 39-bp repeats. Apart from a 39-bp deletion and two single-nucleotide polymorphisms (SNPs), the *orf125* and *orf138* genes share 100% sequence identity with each other, which means that these two genes are highly homologous. Kos CMS caused by *orf125* and Ogura CMS caused by *orf138* can both be fertility restored by the fertility restorer gene *Rfo*/*Rfk* [114]. Kazama et al. (2019) knocked out the mitochondrial gene *orf125* by mitoTALENs in rapeseed, and the hypothesis that *orf125* is the driver of Kosena-type CMS was verified, which strongly suggested that the *orf138* gene is the sterility gene of Ogura CMS [120].

## 3. Mapping and Cloning of Ogura CMS Fertility Restorer Genes in Cruciferous Crops

### 3.1. Ogura CMS Fertility Restorer Genes in Raphanus Sativus

Parallel with the discovery of sterile cytoplasms, FR genes were identified as the genetic means for the recovery of the lost harmony between the mitochondrial and nuclear genomes [121]. To date, all the Ogura CMS FR genes (*Rfo*, *Rfk*, *RF1*, *RF2*, *RF3*, *Rfob*, *Rfoc*, *RsRf3-1*/*RsRf3-2*, *RsRf3-4*~*RsRf3-7*, *Rft* and *Rfs*) have been reported in radish and cloned from European radish, Japanese radish and Chinese radish (Table 2). The Ogura CMS fertility restorer gene *Rfo* was first cloned using map cloning and comparative genomic analysis in radish [51,122]. A fertility restorer gene of Ogura CMS named *Rfk* was cloned and identified as the same gene as *Rfo* [123]. Three populations were used to locate *RF1*, *RF2* and *RF3* in the upper region of the Rsl linkage group, the middle region of the Rs2 linkage group and the upper region of the Rs7 linkage group, respectively, and all three genes showed dominance and were mutually epistatic [124]. The *Rfob* and *Rfoc* genes can also restore the fertility of Ogura CMS radish [52]. *Rfob* is closely linked with the *Rfo* gene and has two SNPs compared with *Rfo*. *Rfoc* is generated by recombination between *Rfo* (*PPR-B*) and *PPR-C*. In addition, several new fertility restorer loci, namely *RsRf3-1/RsRf3-2* and *RsRf3-4~RsRf3-7*, in the Chinese radish materials 2007H, 9802H and 9606H were also cloned and sequenced [53,125,126]. A new restorer gene of Ogura CMS, *Rft,* which was found in the Tomioka population of Japanese wild radish, was also a homolog of *Rfo* and had 19 SNPs compared with *Rfo* [50]. Yamagishi et al. (2021) confirmed that Ogura CMS had a new fertility restorer gene, *Rfs*, which could restore the fertility of individuals by processing *orf138* mRNA and was different from *Rft* [54].

Several intragenic markers or closely linked markers have been developed, such as AFLP markers (R3, R15, R5 and AFLP190), RFLP-PCR markers (2F/1R), RAPD markers (OPA-14_600_ and OPK-17_440_) and STS markers (STS-A14 and STS-K17) [51,122,127]. A high-throughput molecular marker suitable for radish population screening was also designed. Wang et al. (2017) developed a high-throughput SNP marker named Rfo-SNP1 based on the nucleotide sequences of *Rfo* in radish using KASP technology, which has been successfully applied in the high-throughput screening of radish restorer lines [128] (Table 2).

### 3.2. Ogura CMS Fertility Restorer Genes in Other Cruciferous Crops

With the wide application of Ogura CMS, fertility restorer genes have been successfully introduced into different cruciferous crops and integrated at different genomic locations. In *B. napus*, three main fertility restorer genes in Ogura CMS have been localized and cloned, namely *Rfo*, *Rfob* and *Rft* [91,118,129,130]. They were integrated into the C genome (C09, C03) or A genome in different *B. napus* materials. The *Rfo* restorer gene in the restorer line R2000 created by INRA and the restorer line 46H02 created by Pioneer Hibred were both located in the N19 linkage group, and a large number of gene-specific markers and linked markers were developed [129,141]. In CLR650, a new fertility-restored material of Ogura CMS *B. napus*, the restorer gene was located in the N19 linkage group between the SSR markers BnGMS35 and BoGMSl97 [137]. In the Ogura CMS restorer line N1717 of *B. napus*, the *Rfo* gene was confirmed to be located in the C genome by bacterial artificial chromosome–fluorescence in situ hybridization (BAC-FISH), and it was inferred to be located on chromosome C03 according to the size of the chromosome and the location of the centromere [129]. In a new version of the Ogura CMS restorer line CLR6430, Wang et al. (2020) detected the restorer gene by FISH using the C genome and BAC as a probe and verified the presence of *Rfo* in the A genome of *B. napus* [130]. In *B. rapa*, the Ogura CMS fertility restorer gene was integrated into an additional radish chromosome. *Rfk1* of Kosena CMS is a homolog of the fertility restorer gene *Rfo* of Ogura CMS. It was transferred from *B. napus* to Ogura CMS *B. rapa*, and its location on the chromosome was identified by BAC-FISH technology [138]. In *B. juncea*, the *Rfo* gene was located in an ~108 kb radish chromosome fragment, which was positioned amidst a large C-genome translocation at the distal part of chromosome A09, and an intragenic KASPar marker (KASP-RFO-1814) for the marker-aided transfer of *Rfo* was developed [99,140].

## 4. Mechanisms of Ogura CMS in Major Cruciferous Crops

**Figure 2:**
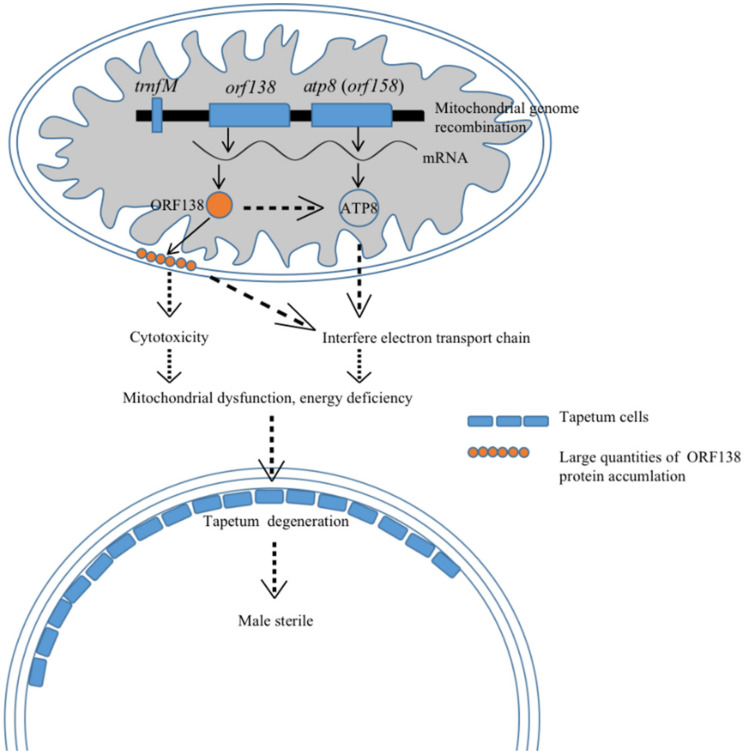
Model of the mechanisms of the Ogura CMS fertility restorer gene *Rfo* (*PPR-B*).

The factor that most directly causes MS in plants is abnormal pollen development [142]. Although the detailed process by which sterility genes affect floral organ and pollen development is still unclear, it is generally believed that mitochondrial dysfunction is mainly responsible for the disruption of pollen development [6,143]. Furthermore, it was shown that during the development of pollen grains, the mitochondrial content of tapetum cells was 40 times that of somatic cells, and the mitochondrial content of microspore cells was 20 times that of somatic cells. Therefore, anthers require more ATP and are extremely sensitive to changes in mitochondrial ATP synthesis [144]. The *orf138* gene encodes a mitochondrial transmembrane protein, similar to many other CMS genes [145]. In sterile plants, the ORF138 protein accumulates largely on the mitochondrial membrane, which may interfere with the expression of key genes in the electron transport chain, such as *ATP6*, *ATP8* and *COXI*, and inhibit anther synthesis (Figure 2). In addition, the occurrence of CMS is mostly related to some chimeric ORFs generated by mitochondrial genome rearrangement [146]. Tanaka et al. (2012) found that the Ogura CMS cytoplasm may be generated by mitochondrial genome rearrangement, and this rearrangement is likely to destroy mitochondrial structures, thereby resulting in Ogura CMS [20].

The abnormal development of tapetal cells was confirmed as an important cause of abnormal pollen development. Normal microsporogenesis requires the appropriate timing of tapetum degeneration [147]. Studies revealed that pollen abortion in Ogura CMS occurred after the tetrad stage [147]. Lin et al. (2019) found that aberrant anther development occurs during the transition from microspore mother cells to tetrads, and defective microspore development and the early clearing of tapetal cytoplasm led to shrunken anthers with collapsed locules. Some studies indicated that the autolysis process, rather than the normal programmed cell death (PCD) process, led to the premature death of tapetal cells, which hindered the development of pollen in the microspore stage of large vacuoles and finally led to MS [55,148]. In addition, the abnormal proliferation of tapetal cells was confirmed as an immediate cause of Ogura CMS in cabbage [113]. Combining cDNA-AFLP with microarray analysis, only one of twenty-nine AT hook nuclear localized (AHL) family genes, *BoMF2*, was differentially expressed in the anthers of Ogura CMS cabbage and might regulate tapetum proliferation during anther development [149].

Flavonoids play an important role in plant pollen development. Many studies in recent years have shown that the chalcone synthase (CHS) gene is related to MS. Related studies have mainly focused on petunia, maize, rice and other plants [147,150]. In radish, the expression of CHS in the flavonoid biosynthesis pathway of Ogura CMS anthers was reduced, and the decrease in CHS expression led to a decrease in flavonoid content, which may ultimately have a negative impact on the production of pollen grains [55].

The possible toxicity of ORF138 remains unclear, and direct evidence for a cytotoxicity model is still lacking. The ORF138 protein was expressed in almost all tissues, including hypocotyls, leaves, roots and buds, but its expression levels varied among the tissues. In vegetative tissues, no morphological or respiratory defects were detected, confirming that ORF138 can cause complete pollen abortion without affecting vegetative development and female gametogenesis in Ogura CMS plants [151]. However, the expression of ORF138 in *Escherichia coli* can inhibit normal growth, indicating that ORF138 has a toxic effect on cells [152]. In addition, the expression of *orf138* in the nucleus was realized by fusing and transforming the CMS gene, green fluorescent protein (GFP) and a mitochondrion-targeting peptide into *A. thaliana* and yeast. The *orf138* gene changed the morphology of mitochondria in yeast and *Arabidopsis* transgenic lines but did not inhibit the respiration and growth of yeast and did not cause sterility in *Arabidopsis* [153].

Great progress in high-throughput sequencing, transcriptomics, proteomics and degradomics has provided a large amount of genetic data and unprecedented opportunities to identify differentially expressed genes (DEGs) that play important roles in CMS [6,154,155,156,157,158]. By comparing the Ogura CMS sterile line and the corresponding maintainer line, a large number of DEGs were identified (Table 3). Substantial numbers of DEGs have been shown to be involved in energy metabolism, which underpins the idea that insufficient energy supply may lead to MS [113,159,160,161,162,163,164,165,166]. Microsporocytes give rise to pollen via meiosis, and somatic cells, particularly tapetal cells, are essential for the normal development and release of pollen [167]. The failure to produce functional pollen in Ogura CMS lines is found to be accompanied by DEGs involved in microspore formation, anther development, pollen wall formation, exine formation (the formation and dissolution of the callose wall, fatty acid metabolism pathway and biosynthesis of sporopollenin precursors in the tapetum), the accumulation of the pollen coat and pectinesterase activity [159,160,161,163,164,165,166,167,168]. CMS always induces the expression of stress-related genes, especially HSP genes [160,162,164,168,169]. ORF138 may affect redox status in Ogura CMS plants, although only DEG evidence exists [159,160,164]. Caspase-like and metacaspase activity genes involved in cell apoptosis were also discovered among DEGs, suggesting that pollen abortion in Ogura CMS was related to PCD [164,165]. In addition, the plant hormone auxin plays a central role in plant growth and development [170]. Studies showed that the delayed expression of most auxin-related genes may have caused short filaments and reduced plant growth in Ogura CMS plants [160,171]. DEGs were also enriched in the mitochondrial retrograde signaling pathway [159]. Two novel miRNA/target cascades (novel-miR-335/H^+^-ATPase and novel-miR-448/SUC1) may participate in the communication between the mitochondria and nucleus [172]. Genes that play important roles in the plant defense response were significantly upregulated in Ogu CMS lines, suggesting their likely involvement in Ogu CMS pollen abortion [163,164,171]. Although these -omics technology methods have been extensively applied in DEG exploration in cruciferous crops, the molecular mechanisms underlying Ogura CMS remain elusive. Furthermore, most impacts of *orf138*/ORF138 were primarily based on speculations, lacking direct experimental evidence to support such conclusions.

## 5. Mechanisms of Ogura CMS fertility Restorer Genes in Major Cruciferous Crops

The *PPR-B* gene, but not the *PPR-A* and *PPR-C* genes, restored the fertility of the Ogura CMS materials. Ogura CMS is regulated by the *orf138* gene, and fertility can be restored when the *Rfo* gene is present [51,122]. The *Rfo* gene is present as three highly similar genes: *PPR-A*, *PPR-B* and *PPR-C*. Three homologous copies are arranged in a series, encoding highly similar proteins [51,57,122,123,175]. *PPR-A* and *PPR-C* do not have fertility restoration ability and have no effect on the synthesis of sterility proteins, while the *PPR-B* gene plays a role in the fertility restoration of Ogura CMS materials [57].

Several important functional sites and binding sites of the fertility restorer gene *Rfo* and the sterility gene *orf138* have been identified. Most restorer genes encode pentatricopeptide repeat (PPR) proteins, and all identified Rf-PPR genes evolved from a unique subset of the PPR gene family called the Rf-like or RFL gene family [176,177]. PPR proteins are characterized by tandem repeats of 35-amino-acid motifs, most of which are considered sequence-specific RNA-binding proteins, and they regulate mitochondrial and chloroplast gene expression through editing, splicing and cleavage [178,179,180,181]. In the fertility restoration process of Ogura CMS, all PPR-B repeats are indispensable for complete fertility restoration [182]. An allele sequence analysis of the fertility restorer gene revealed four substituted amino acids (the 118th, 153rd, 170th and 171st amino acids) in the second and third repeats of the PPR, suggesting an essential role in the fertility restoration of these domains formed by these repeats in the *Rfo* gene-encoding protein ORF687 [123]. Yamagishi et al. (2021) and Imai et al. (2002) also considered that the 118th and 153rd amino acids in ORF687 play a crucial role in fertility restoration [54,183]. By comparing the transcript sequences of *rfo*/*rfo* and *Rfo*/*Rfo* homozygote plants, it was found that four amino acids (the 176th to 179th amino acids) of the *Rfo* gene were essential, and the deletion of these four amino acids in the central region of the *Rfo* gene-encoding protein reduced fertility restoration ability [182]. In the interaction of a nuclear fertility restorer gene and a mitochondrial CMS gene, Uyttewaal et al. [57] proposed that ORF687 could associate with the 5’ untranslated region (5’ UTR) of *orf138* mRNA. Yamagishi et al. (2021) found that direct ORF687 binding to the coding region of *orf138*, not the 5’ UTR inferred previously, was essential for fertility restoration by *Rfo* [54]. It was also found that either a single nucleotide substitution (the 61st nucleotide) in *orf138* or two amino acid substitutions (the 118th and 153rd amino acids) in ORF687 will prevent the *Rfo* gene from restoring the fertility of Ogura CMS materials with the *orf138* gene [54]. Wang et al. (2021) demonstrated that *PPR-B* bound within the coding sequence of *orf138* and found that the GTAAAGTTAGTGTAATA sequence of the *orf138* transcript was the *PPR-B* binding site [184]. Interestingly, the 61st nucleotide was in the binding site, which confirmed the validity and reliability of this conclusion.

**Figure 3:**
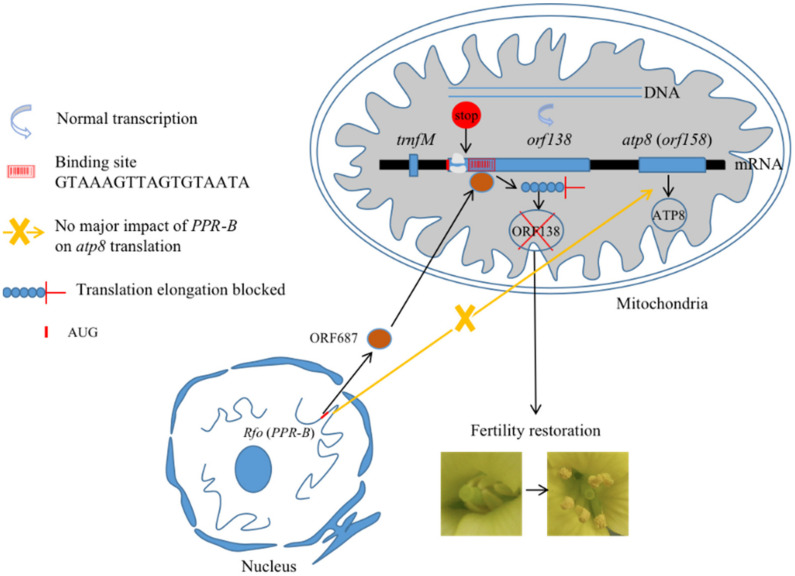
Model of the mechanisms of the Ogura CMS fertility restorer gene *Rfo* (*PPR-B*) (Wang et al., 2021).

The Ogura CMS restorer gene acted at the translation level and restored fertility by blocking the translation elongation of *orf138* mRNA. The mechanisms of different restorer genes are diverse among crops. Fertility restoration in CMS plants is mainly regulated at the genomic level, post-transcriptional level, translational level, post-translational level and metabolic level [6,63]. The function of Ogura CMS restorer genes is generally considered to involve the processing and editing of sterility-related gene transcripts at the post-transcriptional or translational level and then the inhibition of the accumulation of ORF138 and the elimination of the negative effects of sterility proteins on pollen or tapetum development [6,184]. The ORF138 protein showed a significant decrease in the mitochondria of flowers and leaves after the fertility of Ogura CMS *R. sativus* was restored. However, the presence or absence of fertility restorer genes did not affect the size, abundance or RNA editing pattern of *orf138* transcripts, which suggested that the Ogura CMS fertility restorer gene affects the translational level of the *orf138* gene [6,18,57]. Immunolocalization experiments showed that fertility restoration was related to the complete elimination of the ORF138 protein in the tapetum. Therefore, *PPR-B* restores fertility mainly by inhibiting the synthesis of the ORF138 protein in the anther tapetum. Previous research suggested that the precursor RNA of the *orf138* gene in sterile plants was stable because of the formation of a ‘neck-loop’ structure during the splicing process, and it was degraded during the splicing process because of the formation of an unstable 3′ end in fertile plants [185,186]. Therefore, it was concluded that the restorer gene *Rfo* acts post-translationally on the stability of the Ogura CMS-associated protein ORF138 in the reproductive tissues of rapeseed cybrids. By using a restored rapeseed transgenic line containing four copies of *PPR-B*, Wang et al. (2021) confirmed that ORF138 disappeared from mitochondria in the presence of *PPR-B* and rejected the hypothesis regarding a fertility restoration mechanism for the increased instability of ORF138. Wang et al. (2021) demonstrated that this specific translational inhibition of *orf138* mRNA and ORF687 acted as a ribosome blocker to specifically impede translation elongation along the *orf138* mRNA by in organello synthesis, polysome sedimentation and Ribo-Seq analyses [184]. Based on the results of Wang et al. (2021) and other studies, a schematic diagram of the mechanisms of Ogura CMS fertility restorer genes is shown in Figure 3. In addition, another Ogura CMS fertility restorer gene (*Rft*) found in most Japanese wild radishes can affect the expression of *orf138* mRNA [184]. This finding showed that the *Rf* gene, with different molecular mechanisms, evolved in radish and inhibited the expression of the sterility gene *orf138* in Ogura CMS lines.

## 6. Application of Ogura CMS and Creation of Ogura CMS Fertility Restorer Lines

The Ogura cytoplasm has been introduced into different *Brassica* crops, including *B. napus* (AACC), *B. oleracea* (CC), *B. rapa* (AA) and *B. juncea* (AABB), by intergeneric hybridization, somatic hybridization and repeated backcrossing. In rapeseed, it is essential for the *Rfo* restorer gene. In vegetables, because the leafy organ is used as a product for consumers, it is not necessary for fertility restoration to be possible in the vegetative growth stage. However, the wide usage of Ogura CMS has led to a new problem: all offspring not carrying the fertility-restored gene exhibit MS, which has inhibited germplasm innovation and breeding. Therefore, there is an urgent need to create a fertility restorer to promote the innovation and reutilization of germplasm resources in all Ogura CMS *Brassica* vegetables. Corresponding restorer lines have also been successfully created in crops such as *B. napus* (AACC), *B. oleracea* (CC) and *B. juncea* (AABB).

### 6.1. Brassica Napus

The Ogura CMS cytoplasm was successfully transferred to *B. napus*, and the whole process consisted of two stages. (1) The French scholar Bannerot introduced the nucleus of European oilseed rape into Japanese radish sterile cytoplasm and transferred the Ogura CMS cytoplasm into *B. napus* through continuous backcrossing [63]. However, due to cytonuclear discordance, which is a hallmark of many introgression events, many undesirable traits, such as young leaf chlorosis under low temperature and poor nectary development, affect normal growth and development [58,64]. (2) In 1983, Pelletier successfully transferred Ogura CMS to *B. napus* by protoplast fusion and solved existing problems in the previously created Ogura CMS *B. napus*. The male-sterile line exhibited normal green leaves at 12 °C, the normal development of nectaries and the stable inheritance of sterility [65]. Ogura CMS lines of *B. napus* were also introduced in China, and massive restorer line screening was performed [66,67,68]. Li et al. (1995, 2001) introduced the FR gene into *B. napus* by hybridization with the radish variety ‘Makino’ and finally gained the addition line Ad-6, which unfortunately could not be applied in hybrid seed production because of unstable fertility and poor agronomic traits. Chen et al. (2012) used grafting technology to overcome the reproductive obstacles of distant hybridization. Using an interspecific doubling hybrid of oilseed rape and radish (AACCRR), the restorer line CLR650 with high glucosinolate content was selected after multigeneration backcrossing and screening, but it still needs to be improved [69]. Wen et al. (2010,2016) obtained the restorer line R2008 by multigeneration backcrossing, test-crossing and microspore culture and further improved it to obtain six restorer lines with better agronomic traits [68,70].

The creation and improvement of Ogura CMS *B. napus* restorer lines have undergone 30 years of development, and the resulting lines have already been applied in commercial production. Through a large number of screenings, scientists found that there are no natural fertility restorer lines of Ogura CMS in *Brassica* species, and the restorer genes in radish must be transferred to *Brassica* species by intergeneric hybridization. The creation of a fertility restorer line for Ogura CMS in *B. napus* can be divided into three stages. (1) Fertility-restored interspecific materials were first obtained by hybridization and/or protoplast fusion between *R. sativus* with a restorer gene and *B. napus* [65,71]. However, due to the influence of large redundant radish fragments, *B. napus* fertility restorer lines have poor agronomic traits, low seed setting rates and even high glucosinolate content under the influence of close linkage between glucosinolate genes and the restorer gene *Rfo* [127]. (2) With the realization of marker-assisted selection (MAS) in many crops and the development of *Rfo*-linked markers, the improvement of restoration materials has been greatly accelerated [72]. MAS combined with backcross breeding was used to obtain low-glucosinolate-content restorer lines, but some redundant genes closely linked to *Rfo* were still retained in the created materials, resulting in poor agronomic traits [73]. (3) INRA finally provided the restorer line R2000 with both low glucosinolate and low erucic acid levels by combining gamma-ray irradiation with multigeneration backcross breeding [74]. Gamma-ray irradiation greatly increased the genome recombination rate, and the improved fertility restorer line had good agronomic traits, a normal seed setting rate and good *Rfo* transmission efficiency, which have been successfully applied to seed production in Ogura CMS *B. napus*. Pioneer Hi-Bred International Inc. screened a series of *Brassica* mutants with reduced radish fragments from tens of thousands of plants obtained by gamma-ray irradiation and finally bred a new Ogura CMS restorer line (SRF line) of *B. napus* with fewer radish fragments and a lower glucosinolate content than R2000 [75].

### 6.2. Brassica Oleracea

Ogura CMS was transferred into cabbage by distant hybridization between radish and cabbage combined with embryo rescue, and OguraR_1_ CMS cabbage was created [58]. However, because the obtained Ogura CMS cabbage has a radish cytoplasm, problems frequently occur, such as nuclear–cytoplasmic incompatibility, chlorosis at low temperature (15 °C), nectary dysplasia and poor seed set. OguraR_2_ CMS materials were successfully obtained by replacing the radish chloroplast in the OguraR_1_ CMS system with the cauliflower chloroplast by protoplast fusion [77]. Most likely because of the large proportion of the radish cytoplasm in protoplast fusion plants, the growth vigor of two sterile lines derived from OguraR_2_ CMS materials decreased, while the numbers of abnormal flowers and pods increased after 5–6 generations of transformation breeding [78]. The Asgrow seed company applied the asymmetric protoplast fusion method to reduce the proportion of radish mitochondria and obtained Ogura CMSR_3_ with normal sterility and pistil structure. With the Ogura CMSR_3_ material as the sterile source, several Ogura CMS lines of cabbage with stable sterility and normal flowering and seed set have been bred and widely used in the production and breeding of cabbage hybrids. A number of new varieties have been approved or identified, such as ‘Zhonggan 22’, ‘Zhonggan 192’, ‘Zhonggan 96’ and ‘Zhonggan 101’ in China [79].

The *Rfo* restorer gene was introduced from *B. napus* to *B. oleracea* by the bridge material *Brassica alboglabra* Bailey [80,81,82]. Using triploid and hexaploid methods, several Ogura CMS fertility-restored plants of *B. alboglabra* Bailey were successfully created. A series of fertility-restored cabbage plants in the BC_5_ generation were obtained by crossing the BC_2_ generation of *B. alboglabra* Bailey fertility-restored plants with cabbage materials, and these materials were successfully used for the fertility restoration of Ogura CMS clubroot resistance resources in *B. oleracea* [83]. Li et al. (2021) produced the Ogura CMS *Rfo* restorer by transforming the modified *Rfo^B^* restorer gene into the Ogura CMS line ‘CMS2016’ of *B. oleracea* var. *capitata* and developed 18 different morphological Ogura CMS restorers [84]. The polyploid materials, introgressive lines and interspecific hybrid materials created from the above distant hybridization approaches will also provide important materials for several essential research topics, such as polyploidization, distant hybridization, the influence of alien fragments, the innovation of gene function and the formation of important traits.

### 6.3. Brassica Rapa

Ogura CMS was successfully transferred into Chinese cabbage [91,92]. Williams and Heyn (1981) introduced the sterility gene from Japanese radish into Chinese cabbage and bred many male-sterile lines of Chinese cabbage [92]. However, there were different defects in these germplasm resources after the screening of Ogura sterile lines. Delourme et al. (1994) also reported the transformation breeding of the Ogura cytoplasm from *B. napus* to *B. rapa* [91]. In China, sterile resources were collected from the United States, Germany and other countries. Some Ogura CMS Chinese cabbage lines were introduced from abroad, and a number of sterile lines that basically overcame the defects of seedling yellowing, nectary degeneration, bud abortion and a low seed setting rate of the original Ogura sterile lines of *B. rapa* were developed and obtained after continuous multigeneration backcross breeding [93,94,95]. Hou et al. (2001) transferred Ogura CMS into Chinese cabbage by an asymmetric fusion technique [96]. Cui et al. (2004) used Ogura CMS *B. napus* as the female parent and inbred lines as the recurrent parent to conduct hybridization and continuous backcross breeding and finally obtained three CMS lines of *B. rapa* with stable sterility and nonchlorosis [97]. Zhao et al. (2007) introduced the sterility of RC97-1 into Chinese cabbage by using Ogura CMS *B. napus* RC97-1 as a sterile source and then bred a new Ogura CMS line, RC7, which overcame many shortcomings of the original Ogura CMS material through continuous backcrossing and strict economic trait selection [98]. Although Ogura CMS Chinese cabbage has been developed over a long period, it is generally believed that the lack of excellent Ogura CMS Chinese cabbage varieties with good economic traits and large-scale production is due to the problems of disease resistance, poor combination ability, low yield, negative cytoplasmic effects and the severe degradation of backcross generations despite some progress in addressing the problems of yellowing and nectary development. In addition, restorer lines of *B. rapa* have not been created or reported.

### 6.4. Brassica Juncea

CMS lines of *B. juncea* are obtained in three main ways: (1) spontaneous CMS; (2) CMS created by introducing the *B. juncea* nucleus into a heterologous cytoplasm, with the resulting CMS given a name that reflects the species, such as Moricandia CMS [100], Trachystoma CMS [101] and Siifolia CMS [102]; and (3) CMS, first widely used in *B. napus*, transferred into *B. juncea* through protoplast fusion, somatic hybridization and conventional backcrosses, such as Tournefortii CMS [103], Oxyrrhina CMS [104] and Ogura CMS [62]. Delourme et al. (1994) used protoplast fusion to transfer Ogura CMS from *B. napus* to *B. juncea*. Kirti et al. (1995) also reported the transfer of Ogura CMS to the *B. juncea* variety RLM198 by using continuous backcrossing combined with variety screening. However, the obtained sterile line exhibited serious defects, such as seedling yellowing, late flowering time, poor seed set and small or abnormally shaped pods. Therefore, Kirti et al. (1995) further fused the protoplast of the Ogura sterile line and normal cytoplasmic variety RLM198 and obtained an improved Ogura CMS line with great improvement in the above defects by continuous backcrossing. INRA also created the Ogura sterile line and restorer line of *B. juncea*, and Tian et al. (2014) introduced it in Canada [99]. The sterile line was proven to be stable, while the introduced Ogura CMS restorer line in *B. juncea* had a low seed setting rate and many unfavorable agronomic traits, preventing the restorer line from being used. Using MAS combined with backcross breeding, Tian et al. (2014) successfully developed a homozygous Ogura CMS restorer line with a high seed set and good agronomic traits in *B. juncea*, and the linkage redundancy of the improved restorer line was greatly reduced [99].

### 6.5. Other Cruciferous Crops

In *Brassica campestris*, an Ogura CMS line was obtained by crossing the Ogura CMS line of *B. napus* with *B. campestris* and backcrossing with the original male parent [92].

In *B. campestris* L. var. *purpurea* Bailey, a corresponding CMS line was obtained by using Ogura CMS material in *B. napus* after three consecutive years of backcrossing [105]. Since this variety is grown for its consumable vegetative organs rather than its seeds, there is no need for restorer lines.

In *B. oleracea* L. var. *botrytis* L., an Ogura CMS line with resistance to atrazine herbicide was obtained by protoplast fusion technology [85]. Jiang et al. (2001) used Ogura CMS cauliflower introduced from the United States as the female parent to improve Ogura CMS cauliflower in China [86]. The sterile line bred by Jiang et al. exhibited normal growth and development, better disease resistance, good agronomic traits and a good combination ability. Two excellent cross combinations were developed for demonstration and promotion in production. Dey et al. (2011) developed and characterized an Ogura-based improved CMS line of cauliflower [87]. Shu et al. (2019) evaluated the effects of different CMS sources on the morphology and size of floral organs, the attractiveness to pollinators and the yield of hybrid seeds and selected four CMS sources (including Ogura CMS) suitable for future use in broccoli breeding [187]. Singh et al. (2021) characterized a large genetic stock of cauliflower encompassing 76 CMS (consisting of 67 Ogura cytoplasm) and DH lines and laid a good foundation of F_1_ hybrids creation through the effective utilization of CMS and doubled-haploid (DH) lines in cauliflowers [188].

In *B. oleracea* var. *italica* Plenck, He et al. (1999) overcame the defects of pistil dysfunction, bud closure, bud abortion, stigma exsertion and nectary deletion in Ogura CMS donors when the donors were backcrossed with broccoli [88]. Zhu et al. (2001) used the Ogura CMS line 92-08 as the female parent and different broccoli types as the male parent to breed the sterile line BC7-19, which basically overcame the defect of yellowing at low temperatures in the seedling stage [89]. The flower organ structure was normal, and the seed setting ability was strong. Using this broccoli sterile line, the first-generation hybrid ‘Luqing No. 1’ of broccoli was prepared and had the advantages of strong growth potential, early maturity, excellent comprehensive traits and significant yield benefits. Using Chinese kale 16Q2-11 containing the fertility restorer gene *Rfo* as an intermediate material, the *Rfo* gene was introduced into six Ogura CMS broccoli lines by hybridization and subsequent backcrossing [90].

## 7. Discussions

### 7.1. How Do CMS Sterility Genes and Fertility Restorer Genes Come about?

Plant mitochondrial genomes are rich in repeated sequences, with estimates of up to 38% of the mitochondrial genome occupied by tandem repeats, short repeats and large repeats [189,190]. Repetitive sequences are the most common and important sequences in the mitochondrial intergenic region. They are related to the mutation and recombination of the mitochondrial genome and play an important role in the evolution of plant mitochondrial genomes. The comparison of the mitochondrial genomes of CMS lines and maintainer lines showed that there were almost no deleterious mutations in CMS line genomes except that of *Beta vulgaris* ssp. *maritima* with G-type CMS, which means that deleterious mutations are not the root cause of CMS [191,192]. Together with the reported mitochondrial genome sequences, comparative analysis showed that most CMS types are closely related to repeating sequences. Heng et al. (2014) found that repeats in the CMS line were typically twice the size as those in the maintainer line and three large repeats were present downstream of the hau CMS-associated gene *orf288*, which implicated the strong ties between repeat sequences and the formation of this chimera [193,194]. Comparative analysis between the mitogenomes of CMS and male-fertile lines of pepper (*Capsicum annuum* L.) showed that the CMS candidate genes *orf507* and *ψatp6-2* were proximal to the edges of highly rearranged CMS-specific DNA regions, whose evolution may be the result of nearby intermediate or large-sized repeats [195]. Tanaka et al. (2014) reported that the Ogura-type mitochondrial genome was highly rearranged compared with the normal-type genome by recombination through one large repeat and multiple short repeats. A high rearrangement rate is a common feature of CMS mitochondrial genomes. Recombination via repeat sequences is believed to be responsible for extensive structural changes [20]. The structure of plant mitochondrial genomes evolves rapidly, driven by rearrangements that result from high rates of recombination [196]. Thus, CMS leading to mitochondrial genome rearrangement may be associated with DNA recombination mediated by repeating sequences and probably arose in progenitor species, which was then revealed when hybridization removed the corresponding nuclear restorer gene. Mutation and selection are two primary forces that drive evolutionary processes [197]. Any nuclear allele able to restore the function of the male gametophyte in plants with sterility genes will be favored and subsequently fixed in a population. As the frequency of the CMS gene increases in the population, selection will more strongly favor the restorer alleles, which should subsequently spread in the population.

### 7.2. What Are the Future Development Directions of Ogura CMS?

Ogura CMS is the most extensively studied and widely used CMS system in Brassicaceae crops [54]. Nevertheless, many open questions remain, and this situation has changed in recent years with the development of new techniques. According to the limitations and problems during the application of Ogura CMS in existing research, future directions are discussed and then summarized into two major aspects.

More direct evidence for the functional gene validation of the sterility gene *orf138* is still needed. Although the overexpression of the *orf138* gene in transgenic *Arabidopsis* and the knockdown of the *orf125* gene, which is the homologous gene of *orf138*, have been achieved, functional genomics research needs to employ diverse experimental approaches to investigate gene functions [198]. Gene editing of *orf125* was implemented by using transcription activator-like effector nucleases (TALENs) with mitochondrial localization signals (mitoTALENs). This approach has also been applied to validate the mitochondrial gene *orf312* of Tadukan-type CMS and the mitochondrial gene *orf79* of CMS-BT in rice [120,199]. CRISPR-based gene editing has revolutionized targeted gene editing in plants in recent years [200]. CRISPR-mediated genome editing was realized in the mitochondria of yeast and the chloroplasts of Chlamydomonas [201]. Kang et al. (2021) achieved DNA-free editing in chloroplasts and generated lettuce calli and plantlets resistant to streptomycin or spectinomycin [202]. The Northwest Institute of Plateau Biology, Chinese Academy of Sciences, also unveiled a method to develop male-sterile lines in plants using a mitochondrial gene editing system [203]. One of the most direct pieces of evidence would be to verify the function of the *orf138* gene by editing ORFs related to CMS with mitochondrial gene editing to silence the *orf138* gene and directly realize fertility conversion. In addition, the transcription-specific targeting of mitochondrial gene expression is difficult, and it is also necessary to realize the mitochondrially targeted expression of the *orf138* gene in Cruciferae crops, except for *Arabidopsis*.

A great deal remains to be determined about sterility and fertility restoration mechanisms. (1) The mechanism by which sterility genes affect pollen abortion but not female gametes and other tissues remains unclear. Research on CMS-S in maize has led to a breakthrough in solving this problem. Xiao et al. (2020) found that the nuclear-encoded DREB transcription factor *ZmDREB1.7* was specifically expressed in anthers and promoted the expression of the CMS-S gene *orf355* [204]. The accumulation of ORF355 activated mitochondrial retrograde signaling and in turn induced *ZmDREB1.7* expression. *ZmDREB1.7* and *orf355* formed a positive-feedback transcriptional regulation loop in microspores of CMS-S maize, which eventually led to the accumulation of the ORF355 protein and abortion. Similar transcription factors may also exist in Ogura CMS and influence pollen abortion without affecting other tissues. Recent advances in single-cell sequencing technologies would be useful for addressing this issue. Zhang et al. (2021) isolated the anthers of CMS lines and restorer lines, analyzed their transcriptional expression profiles by single-cell RNA sequencing, and then expounded the mechanism of CMS-C sterility genes and nuclear restorer genes affecting meiosis and microspore development [205]. There are still many research gaps in the genetics, cytology and molecular biology fields related to male and female gametophyte development. Breakthroughs in future leading-edge technology are expected to provide insights into the regulatory mechanism of male and female gametophyte development at the single-cell level and into basic theoretical research on sterility and fertility restoration mechanisms. (2) Problems such as large differences in pollen amount and pollen viability and a low *Rfo* gene transmission rate still exist in fertility restorer lines [83]. Su et al. (2016) performed bulked segregant RNA-Seq and identified six potential associated genes in minor effect QTLs contributing to fertility instability, which inspired us [206]. Studies have shown that the introgression of exogenous fragments has an effect on genetic variation in chromosomes possessing chromatin fragments and may result in both whole-genome shock and local chromosomal shock [207,208]. The effect of exogenous fragments from *R. sativus* and *B. napus* will be an important direction for future research on the Ogura CMS/FR system.

## Figures and Tables

**Table 1:** Sequenced mitochondrial genome information for Ogura CMS sterile lines and corresponding maintainers in cruciferous crops.

Cruciferae Crop	Material	Genome (bp)	Accession No.	Genes	Unique Regions	Reference
*Raphanus sativus*	Ogu CMS maintainer	244,036	AB694743	33 protein-coding genes, 3 rRNA genes and 17 tRNA sequences	/	[20]
*Raphanus sativus*	Ogu CMS	258,426	AB694744	Same genes with its maintainer line and additional *atp9* and tRNA gene *trnfM*	Four	[20]
*Brassica oleracea*	Ogu CMS maintainer	219,962	MW423604	31 known genes, 67 ORFs, 3 rRNA genes and 25 tRNA genes	/	[113]
*Brassica oleracea*	Ogu CMS	236,648	MW423605	30 known genes (lost *rps7* gene compared with the maintainer line), 69 ORFs, 3 rRNA genes and 25 tRNA genes	Five	[113]
*Brassica oleracea*	Ogu CMS maintainer	219,969	ON758774	33 protein-coding genes, 3 rRNA genes and 18 tRNA genes	/	[114]
*Brassica oleracea*	Ogu CMS	71,998, 185,431	ON758776, ON758777	Same genes with its maintainer line and additional *orf138* and three tRNA genes (*trnfM-CAU*, *trnN-GUU* and *trnY-GUA*)	Nine	[114]
*Brassica juncea*	Ogu CMS maintainer (RLM198)	219,776	MT675103	/	/	[115]
*Brassica juncea*	Ogu CMS (OgRLM)	258,462	MT675104	/	/	[115]
*Brassica juncea*	Ogu CMS (Og1)	250,999, 96,185	MT675105, MT675106	34 protein-coding genes, 3 rRNA genes and 15 tRNA genes	/	[115]
*Brassica juncea*	Ogu CMS revertant line (Og1-rt)	250,999	MT675105	33 protein-coding genes (lost the *orf138* gene compared with the sterile line Og1), 3 rRNA genes and 15 tRNA genes	/	[115]
*Brassica napus*	Ogu CMS	258,473	Unknown	33 protein-coding genes, 3 rRNA sequences and 23 tRNA sequences	Nine	[116]

**Table 2:** Comparative analysis of Ogura CMS systems in different Cruciferae crops.

Cruciferae Crops	Sterility Gene Markers	FR Genes	FR Gene Loci	Linked FR Gene or Intragenic Molecular Markers	Advantages	Limitations	References
*Raphanus* *sativus*	Primer A/B, CMSF/R, orf138-F/R	*Rfo* (*Rfk*), *RF1*, *RF2*, *RF3*, *Rfob*, *Rfoc*, *RsRf3-1*/*RsRf3-2*, *RsRf3-4*~*RsRf3-7*, *Rft*, *Rfs*	C3	R3, R15, R5, AFLP190, Rfo-SNP1, RFLP-PCR2F/1R with the Ssp1 restriction enzyme site, OPA-14600,OPK-17440, STS-A14, STS-K17	(1) The most studied male sterility type in radish (2) Complete and stable sterility (3) Many FR genes in European radish, Japanese radish and Chinese radish varieties	(1) Low distribution frequency of the rfrf genotype (2) Maintainer line selection requires a large number of backcross offspring	[51,52,53,54,59,60,61]
*Brassica* *napus*	P51/P52	*Rfo*, *Rfob*, *Rft * (introduced from *R. sativus*)	N19, C3, A genome	PGI-2, OPC02_1150_, OPD02_1000_, OPF06_1200_, OPG02_700_	(1) Long history of creation and thorough study in Ogura CMS lines and restorer lines (2) The safest and most effective way to utilize heterosis (3) Three-line breeding and commercial production have been achieved	(1) Large radish alien genome segments around the FR gene and poor agronomic traits of restorer lines (2) Restorer lines and breeding methods with low glucosinolate content have been submitted for long-term patent protection, which greatly limits the application	[20,72,73,129,130,131,132,133]
*Brassica oleracea*	Bo138300BF/R; P1/P2; P12F/R; P13F/R; OKB-F/R	*Rfo* (introduced from *R. sativus*)	/	BnRFO-AS2F/BnRFO-NEW-R, Rfo-6F/R, Rfo-6eF/R, Rfo-8F/R, Rfo-11F/R, Rfo-page-4eF/R	(1) Complete and stable sterility (2) Fertility-restored materials have been created with 18 normal chromosomes, a normal seed setting rate and a closer genetic background to the parent cabbage	(1) Varied fertility restoration levels (pollen vitality) of fertility restorer lines (2) Low and abnormal *Rfo* gene transmission rate (3) Large proportion of radish and rapeseed genomic components	[80,81,82,83,114,134,135,136]
*Brassica* *rapa*	F/R; P11/P12; orf138 primer F/R	* Rfk1 * (introduced from radish and homolog of *Rfo*)	Additional radish chromosome	F/R	(1) Fertility of Ogura CMS can be restored by the *Rfk1* gene	(1) Restorer gene was unstable in the turnip rape genome (2) Fertility restoration trait was unstable in subsequent generations	[137,138,139]
*Brassica juncea*	/	*Rfo* (introduced from *R. sativus*)	A09	KASP-RFO-1814	(1) Good seed set and agronomic performance in restorer lines	(1) A large radish introgression alien segment carrying the *Rfo* gene and linkage drag exist (2) Pollen contamination and impaired transmission frequency of the *Rfo* gene	[99,140]

**Table 3:** Various -omics studies on Ogura CMS in cruciferous crops.

Cruciferae Crops	Materials	Omics Technologies	Functions of DEGs	Reference
*Raphanus sativus*	Sterile line, maintainer line	Transcriptomics	Fatty acid metabolism, pollen development and tapetum development	[56]
	Sterile line, maintainer line	Transcriptomics	Binding, catalytic activity, metabolic process, cellular process and response to stimulus	[162]
*Brassica napus*	Sterile line	Proteomics	Carbohydrate and energy metabolism, aldehyde dehydrogenase (ALDH), photosynthesis and flavonoid synthesis	[161]
	Sterile line, maintainer line	Transcriptomics	Plant hormone signal transduction, plant–pathogen interaction, peroxisome, pentose–glucuronate interconversions and starch–sucrose metabolism	[171]
*Brassica oleracea*	Sterile line, maintainer line	Transcriptomics	Biosynthesis of secondary metabolites, starch and sucrose metabolism, plant–pathogen interaction and glycerophospholipid metabolism	[163]
	Sterile line, maintainer line	Transcriptomics	Polygalacturonase metabolism, glycosyl hydrolases, oxidation reduction process, phenylalanine metabolism and phenylpropanoid biosynthesis	[164]
	Sterile line, fertile line	Transcriptomics and proteomics	Gibberellin-mediated signaling pathways regulating tapetum programmed cell death and sporopollenin synthesis	[165]
	Sterile line, maintainer line	Transcriptomics and proteomics	Sporopollenin synthesis, callose wall degeneration and oxidative phosphorylation	[166]
	Sterile line, maintainer line	Transcriptomics	Energy metabolic pathways	[113]
*Brassica rapa*	Sterile line, maintainer line	Transcriptomics	Mitochondrial retrograde signaling pathway, auxin response, ATP synthesis, pollen development and stress response	[160]
	Sterile line, maintainer line	Degradome analysis, miRNA analysis and transcriptomics	Pollen-development-related genes	[172]
	Sterile line	Transcriptomics	Stress-response genes, mitochondrial protein genes, ascorbic acid biosynthesis and thylakoid protein gene	[169]
	Sterile line, maintainer line	Transcriptomics	Anther development and microspore formation	[159]
	Sterile line, maintainer line	Transcriptomics	Pollen development, carbon metabolism, lipase activity, lipid binding, citrate cycle and oxidative phosphorylation	[173]
	Sterile line, maintainer line	Transcriptomics	Phenylpropane synthesis pathway and glutathione oxidation–reduction	[174]

## Data Availability

Not applicable.
